# Prevalence of Deep Surgical Site Infection After Repair of Periarticular Knee Fractures: A Systematic Review and Meta-analysis

**DOI:** 10.1001/jamanetworkopen.2019.9951

**Published:** 2019-08-23

**Authors:** Grayson R. Norris, Jake X. Checketts, Jared T. Scott, Matt Vassar, Brent L. Norris, Peter V. Giannoudis

**Affiliations:** 1 High Point University, High Point, North Carolina; 2 Oklahoma State University Center for Health Sciences, Tulsa; 3 Department of Orthopedics, Oklahoma State University Center for Health Sciences, Tulsa; 4 Orthopedic & Trauma Services of Oklahoma, Tulsa; 5 University of Leeds, West Yorkshire, United Kingdom

## Abstract

**Importance:**

Surgical management of periarticular knee fractures can be challenging, and adverse outcomes may be severe. Recent literature indicates that the rate of periarticular knee surgical site infection (SSI) may range from 2% to 88% depending on the fracture site.

**Objective:**

To examine the prevalence of deep SSI and the rate of septic arthritis after surgical repair of fractures around the knee.

**Data Sources:**

The electronic databases MEDLINE, Embase, and the Cochrane Central Register of Controlled Trials were searched from their inception to July 1, 2018.

**Study Selection:**

Eligible studies had to specifically report deep SSI rates and include fractures in the distal femur, patella, tibial plateau, or proximal tibia. Risk factors that were associated with increased the risk of deep SSI were also examined.

**Data Extraction and Synthesis:**

This study followed the Preferred Reporting Items for Systematic Reviews and Meta-analyses (PRISMA) reporting guideline. Data were extracted by multiple investigators. Comprehensive Meta-Analysis software was used for the pooling of data, using either random-effects or fixed-effects models, with respect to the degree of statistical heterogeneity present. Data analyses were conducted in October 2019.

**Main Outcomes and Measures:**

The primary outcome was overall prevalence of deep SSI after periarticular knee fracture repair. The secondary outcomes were the overall prevalence of septic arthritis, risk factors associated with deep SSI, and the most commonly cultured bacteria specimens found periarticular knee infections.

**Results:**

Of 6928 articles screened, 117 articles met inclusion criteria and were included in analysis. Among 11 432 patients included in analysis, 653 patients (5.7%) experienced deep SSIs, most commonly among patients with proximal tibia fractures (56 of 872 patients [6.4%]). Among studies that included information on septic arthritis, 38 of 1567 patients (2.4%) experienced septic arthritis. The 2 most commonly reported bacteria were methicillin-resistant *Staphylococcus aureus*, found in 67 SSIs, and methicillin-susceptible *S aureus*, found in 53 SSIs. Sixty-two studies (53.0%) in the sample received a Coleman Methodological Score of poor (<50 points).

**Conclusions and Relevance:**

Deep SSIs occurred in nearly 6% of periarticular knee fracture repairs, and 2.4% of SSIs were associated with septic arthritis. Surgeons managing these injuries should be vigilant when wounds are not pristine. Efforts should be made to elevate the quality of research conducted not only in this subject but also in orthopedic surgery as a whole.

## Key Points

**Question:**

What is the overall prevalence of deep surgical site infection after surgical repair of periarticular knee fractures?

**Findings:**

This systematic review and meta-analysis examined 117 studies including 11 432 patients. Among them, 5.7% of patients experienced deep surgical site infections, most commonly among patients with proximal tibia fractures (6.4%); among 20 studies that reported data on septic arthritis, 2.4% of patients experienced septic arthritis.

**Meaning:**

Surgeons managing periarticular knee fractures should be vigilant when wounds are not pristine.

## Teaser

This systematic review and meta-analysis examines the prevalence of deep surgical site infections after surgical repair of periarticular knee fractures, and the prevalence of septic arthritis, common microorganisms in infection cultures, and covariates associated with surgical site infections.

## Introduction

The goals for treating fractures around the knee include satisfactory restoration of mechanical alignment, anatomical reduction of the articular surface, and stable fixation to allow early motion of the knee.^1^ Managing these fractures can be challenging, and adverse outcomes can include nonunion, malunion, heterotopic ossification, arthrofibrosis, compartment syndrome, and infection among others.^2^

Recent literature indicates that the rate of postoperative surgical site infection (SSI) may range from 13% to 88% for tibial plateau fractures,^2^ 3% to 17% for distal femur fractures,^3^ 2% to 10% for patellar fractures,^4,5,6,7^ and 3% to 45% for proximal tibia fractures.^8^ Although the reported infection rates of periarticular knee fractures are highly variable, understanding the frequency with which infectious adverse outcomes occur is important to orthopedic surgeons for the management and prevention of adverse outcomes, such as unsatisfactory outcomes for the affected patient and possible loss of function in the affected region.^8,9^ Furthermore, patients with SSIs have been found to have higher mortality rates compared with patients without SSIs, as well as extended time spent in the hospital and higher costs of medical care.^10,11^

Although many studies and a few systematic reviews have been conducted to evaluate the prevalence of SSI after repair of distal femur, tibial plateau, proximal tibia, or patellar fractures, to our knowledge, a single systematic review has not been conducted that elucidates the overall magnitude of deep SSIs after surgical management of fractures around the knee as a whole. The purpose of this systematic review was to obtain a more thorough understanding of the prevalence of deep SSIs after the repair of fractures around the knee. We also evaluated the bacteria associated with these infections in the hope of elucidating which bacteria are most commonly associated with periarticular knee deep SSIs. Furthermore, we analyzed risk factors associated with periarticular knee deep SSIs, such as tobacco use, diabetes, sex, compartment syndrome, and type of fracture (ie, open vs closed) using a meta-analytical method.

## Methods

This systematic review and meta-analysis applied and adhered to the Preferred Reporting Items for Systematic Reviews and Meta-analyses (PRISMA) reporting guideline, and the Cochrane Handbook for Systematic Reviews was closely followed. Our inclusion criteria included studies with patients who were ambulatory and had sustained a distal femur, tibial plateau, proximal tibia, or patellar fracture, whether as a single injury or as a component of multiple trauma. All interventions used for these patients were included in our analysis.

Experimental and animal studies, review articles, articles with a primary patient population younger than 18 years, letters to the editor, case reports, cadaveric studies, studies with fewer than 20 patients, and studies evaluating fractures associated with metabolic conditions, paraplegia, periprosthetic fractures, or malignancy were excluded. Our primary outcome was the rate of periarticular knee deep SSIs. Our secondary outcomes included risk factors associated with periarticular knee deep SSIs (ie, smoking, diabetes, sex, compartment syndrome, and type of fracture), types of bacteria associated with periarticular knee deep SSI, and fracture location associated with the greatest risk of periarticular knee deep SSI. The overall prevalence of septic arthritis (as a specific type of deep SSI) was evaluated as a secondary outcome, as it is common practice in traumatology research to report deep and superficial infections without delineating whether the deep infection was or led to septic arthritis.

### Study Identification

The electronic databases MEDLINE, Embase, and Cochrane Central Register of Controlled Trials were searched from their inception to July 1, 2018. Data analyses were conducted in October 2019. We used the following terms and Boolean operators: “(distal femur OR distal femoral OR proximal tibia* OR tibia* plateau OR patella*) AND (fracture OR pin OR screw OR nail OR plate OR plating OR fixation OR ORIF) AND (infection OR sepsis OR septic OR adverse effect OR adverse event OR complication OR incidence OR risk factor).” We did not apply any restrictions on language or country of publication as long as the paper was available in English or was able to be translated into English via Google Translate (Alphabet). Furthermore, we examined reference lists of applicable reviews and the included articles for any applicable articles not returned by our search. We also manually searched for recently published studies by evaluating the electronic databases of applicable orthopedic journals to prevent the possibility of missing recently published literature. Using Rayyan (Qatar Computing Research Institute), a tool for optimizing work flow of systematic reviews, 2 of us (G.R.N. and J.X.C.) independently screened titles and abstracts to identify relevant trials. We used Paperpile (Paperpile) to retrieve and access full texts of the studies that met our inclusion criteria.

### Quality Assessment

We used the modified Coleman Methodology Score (CMS) as presented by Saleeb et al^12^ to evaluate the methodological quality of all primary studies included in our analysis.^13^ The modifications to the CMS by Saleeb et al^12^ were slight changes in syntax and checkpoints to better reflect what would be expected from a well-conducted traumatology study. Because their study also evaluated infection rates postfracture, we decided that using this slightly modified version would be better suited for our study. The CMS is a comprehensive tool to evaluate the methodological quality of surgical studies. Factors evaluated by CMS include study size, mean follow-up, number of surgical procedures, study type, diagnostic certainty, surgical protocol description, postoperative rehabilitation description, description of desired outcomes, description of how outcomes were assessed, and description of subject selection process. These categories are assigned a specific subset of points that can be obtained for each level of methodological quality. For example, in the study type category, randomized clinical trials are assigned 15 points, prospective cohort studies are assigned 10 points, and retrospective cohort studies are assigned 0 points. The CMS has a score range from 0 to 100, with a higher score indicating more robust methodological quality and a lower risk of bias and confounding factors. Scores are broken into 4 categories: (1) excellent (85-100 points), (2) good (70-84 points), (3) fair (50-69 points), and (4) poor (<50 points).

### Data Extraction

Two of us (G.R.N. and J.X.C.) used independent double data extraction to evaluate titles and abstracts of the returned articles. For all studies included after the title and abstract screening, we obtained the full text of the article to evaluate for further inclusion. Disagreements in this stage were mitigated by group discussion between both investigators and with a third investigator (B.L.N.) when needed. Within a predefined Google Sheet (Alphabet), extracted baseline characteristics of the included articles and their outcome data were organized by fracture type. To prevent redundancy or duplication of data, the author names and journal titles were not masked throughout this process.

Consistency of included articles and extracted variables within our results was ensured by predetermined definitions and inclusion criteria. For instance, an SSI was considered a deep SSI if the article described it as *deep*, *septic*, or *osteomyelitis* or if hardware removal was required owing to the SSI. Furthermore, if the SSI was located deep within the fascia or bone and required bone or soft-tissue debridement, then it was considered a deep SSI. We also delineated whether deep SSIs resulted in septic arthritis if a study specified that an SSI was *septic* or *in the joint*. Superficial SSIs were defined as those which only involved superficial tissues and resolved easily with antibiotic treatment and dressing management. For a study to be included in our sample, the authors had to delineate between superficial and deep SSIs. For example, if the study only used the word *infections* but never specifically stated *deep infection* or *superficial infection*, or it did not describe the characteristics of the SSI, then it was not included in our study.

### Statistical Analysis

Risk factors of interest were expressed as proportions (eg, deep SSI, superficial infection, diabetic status, smoking status). Comprehensive Meta-Analysis software (Biostat) was used for pooling data, using either fixed-effects or random-effects models, depending on the degree of statistical heterogeneity present. The Cochran *Q* and Higgins *I*^2^ tests were used to test statistical heterogeneity. For the Cochran *Q* test, statistical significance was set at .10, and for the Higgins *I*^2^ test, statistical significance was set at 50% or greater. The Mann-Whitney *U* test was used for nonparametric comparisons of the median values between groups of interest.

For studies with different comparator groups, binary outcomes were summarized as odds ratios (ORs) with 95% CIs. For outcomes of interest, pooled estimates of effect size were obtained using the Comprehensive Meta-Analysis software. Depending on the degree of heterogeneity, a fixed-effects or random-effects model was used.

## Results

**Figure 1.**
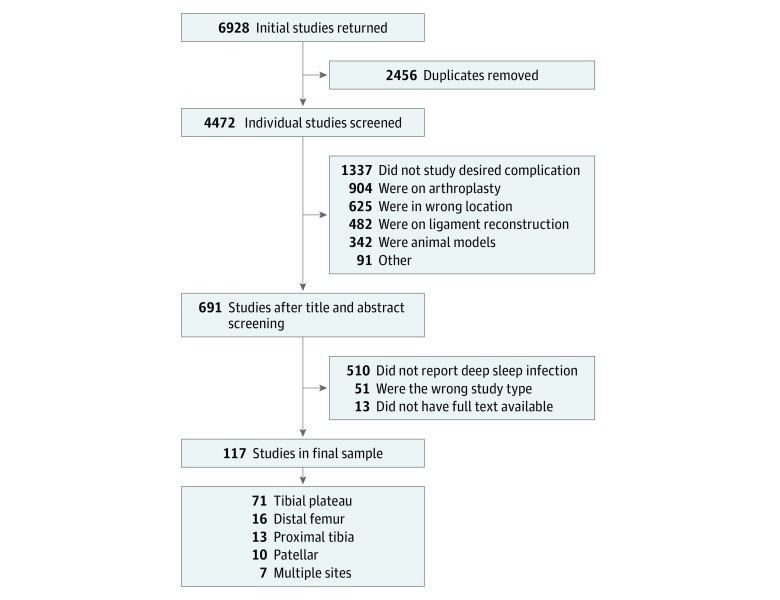
Flowchart of the Selection of Included Studies

Our initial electronic search yielded 6928 results. Following the removal of duplicate studies, a total of 4472 studies were available for title and abstract screening (Figure 1). With our inclusion criteria applied and relevant reference lists screened, 117 studies^1,2,4,5,10,14,15,16,17,18,19,20,21,22,23,24,25,26,27,28,29,30,31,32,33,34,35,36,37,38,39,40,41,42,43,44,45,46,47,48,49,50,51,52,53,54,55,56,57,58,59,60,61,62,63,64,65,66,67,68,69,70,71,72,73,74,75,76,77,78,79,80,81,82,83,84,85,86,87,88,89,90,91,92,93,94,95,96,97,98,99,100,101,102,103,104,105,106,107,108,109,110,111,112,113,114,115,116,117,118,119,120,121,122,123,124,125,126^ with 11 432 patient outcomes (mean [SD] age, 46.6 [6.9] years, range: 28-67 years) were included for this review.

For each primary study, we extracted data regarding design, size, patient demographic characteristics, and outcomes of interest. Of these, 71 studies^2,10,53,54,55,56,57,58,59,60,61,62,63,64,65,66,67,68,69,70,71,72,73,74,75,76,77,78,79,80,81,82,83,84,85,86,87,88,89,90,91,92,93,94,95,96,97,98,99,100,101,102,103,104,105,106,107,108,109,110,111,112,113,114,115,116,117,118,119,120,121^ (60.7%) evaluated tibial plateau fractures, 16 studies^14,15,16,17,18,19,20,21,22,23,24,25,26,27,28,29^ (13.7%) evaluated distal femur fractures, 13 studies^40,41,42,43,44,45,46,47,48,49,50,51,52^ (11.1%) evaluated proximal tibia fractures, 10 studies^4,5,32,33,34,35,36,37,38,39,40,41,42,43,44,45,46,47^ (8.5%) evaluated patellar fractures, and 7 studies^1,30,31,123,124,125,126^ (6.0%) evaluated fractures of multiple sites. Additionally, 81 studies^1,2,4,10,14,19,20,21,22,23,24,27,28,30,31,32,33,34,35,36,37,39,40,41,42,47,49,55,56,60,61,63,64,66,67,68,69,70,71,72,74,75,77,78,79,80,81,83,85,87,88,90,93,94,95,96,97,98,100,101,102,104,106,108,110,111,112,113,114,115,116,117,118,119,121,122,123,124,125,126^ (68.4%) were retrospective, 32 studies^16,17,25,26,29,38,43,44,45,46,48,50,51,52,53,56,57,58,59,62,73,76,82,84,89,91,92,99,103,105,109,120^ were prospective cohort studies, and 4 studies^15,18,24,86^ were prospective randomized controlled trials. Further details of study characteristics can be found in the eTable in the Supplement. Timing to SSI is also described in the eTable in the Supplement; however, because each study described this timing differently, we elected to quote each study’s reported time to SSI because statistics could not be extrapolated.

### Quality Assessment

The CMS score of our included studies ranged from 15 to 97 (mean [SD], 50.41 [15.24]; median [interquartile range], 49 [39-59]). Sixty-two studies (53.0%) in our sample scored a poor CMS score, while 43 studies (36.8%) had a fair CMS score, 10 studies (8.6%) had a good CMS score, and 3 studies (2.6%) had an excellent CMS score. The 3 CMS criteria in which the most studies did not receive points or lost points were the categories evaluating the type of study (most were retrospective), the description of postoperative rehabilitation protocol (most did not discuss this protocol), and the procedure for assessing outcomes (most did not discuss this process).

### Deep SSI Rates

**Figure 2.**
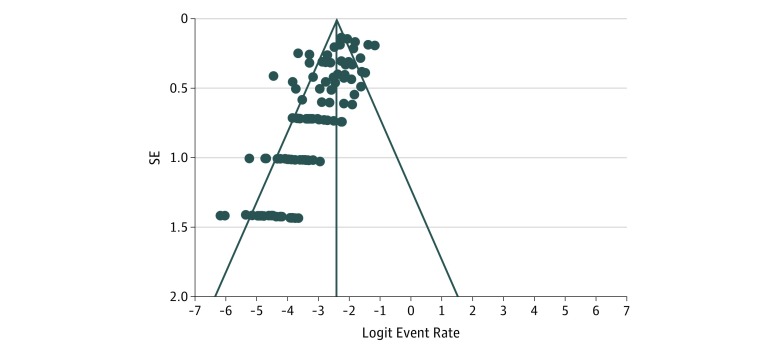
Funnel Plot of Degree of Heterogeneity Within Selected Studies Noticeable asymmetry can be attributed to the number of retrospective studies used.

Among 11 432 patients included in our analysis, 653 (5.7% [95% CI, 4.4%-6.2%]) experienced deep SSIs. Superficial SSIs occurred in 388 patients (3.4%). Figure 2 presents the degree of heterogeneity within the 117 studies. The mean age of the patients did not have a statistically significant association with the deep SSI rate. For studies with a CMS score of excellent or good, the incidence of deep SSI within these studies was 18 of 804 cases (2.2%), whereas the incidence of deep SSI among studies designated as fair or poor was 635 of 10 628 cases (6.0%) (*P* < .001).

#### Distal Femur

There were 1011 distal femur fractures included in our sample. Deep SSIs occurred in 58 patients (5.7% [95% CI, 5.4%-8.8%]), and superficial SSIs occurred in 14 patients (1.4%).

#### Patella

There were 1150 patellar fractures in our sample. Deep SSIs occurred in 47 patients (4.1% [95% CI, 3.9%-6.8%]), and superficial SSIs occurred in 11 patients (1.0%).

#### Tibial Plateau

There were 7925 tibial plateau fractures in our sample. Deep SSIs occurred in 464 patients (5.9% [95% CI, 4.2%-6.3%]), and superficial SSIs occurred in 330 patients (4.2%).

#### Proximal Tibia

There were 872 proximal tibia fractures in our sample. Deep SSIs occurred in 56 patients (6.4% [95% CI, 2.4%-9.7%]), and superficial SSIs occurred in 23 patients (2.6%).

#### Multiple Locations

There were 474 fractures included in studies evaluating multiple areas in our sample. Deep SSIs occurred in 28 patients (5.9% [95% CI, 4.0%-14.1%]), and superficial SSIs occurred in 10 patients (2.1%).

### Septic Arthritis

There were 20 studies that reported septic arthritis among their cohort. These studies included 1567 patients. Among these studies, the septic arthritis occurred in 38 patients (2.4%) (eTable in the Supplement).

### Microbiological Analysis

Sixteen studies^2,26,28,36,41,52,66,72,74,83,84,101,106,111,113,125^ reported the bacterial culture results of the infected fracture sites. The results are listed in Table 1. Of 182 deep SSIs with bacterial culture results, 67 (36.8%) were culture positive for methicillin-resistant *Staphylococcus aureus* (MRSA), and 53 deep SSIs were culture positive for methicillin-susceptible *S aureus*. These were the 2 most commonly reported bacteria.

**Table 1.** Microbiology Culture Results by Study

Source	Fracture Location	Hardware Installed	Culture Findings	Cultures, No.
Phisitkul et al, 2007^41^	Proximal tibia	Plate and screws	MRSA	1
			Bacillus cereus	1
			*Staphylococcus epidermidis* with *Enterococcus* species	1
			*Staphylococcus aureus* with *Haemophilus influenzae*	1
			No growth	4
Cole et al, 2004^52^	Proximal tibia	Plate and screws	MRSA	1
			No growth	1
Kregor et al, 2004^28^	Distal femur	Plate and screws	MRSA	3
Kayali et al, 2007^26^	Distal femur	Plate and screws	MRSA	1
			*Escherichia coli *	1
Barei et al, 2004^66^	Tibial plateau	Plate and screws	MRSA	1
			*Enterococcus* species	2
			*S aureus *	2
			*Enterobacter* species	1
Morris et al, 2013^2^	Tibial plateau	Plate and screws	MRSA	20
			*S aureus *	9
			*Enterobacter* species	9
Momaya et al, 2016^101^	Tibial plateau	Plate and screws	MRSA	26
			*S aureus*	11
			*Enterobacter cloacae *	5
			*Enterobacter faecalis *	5
Lin et al, 2014^106^	Tibial plateau	Plate and screws	MRSA	7
			*S aureus *	8
			*Pseudomonas aeruginosa*	3
			*Enterobacter* species	2
			*Acinetobacter baumannii*	1
			*Enterococcus* species	1
			*Streptococcus* species	1
			*Serratia* species	1
Shah et al, 2007^72^	Tibial plateau	Plate and screws	MRSA	1
			*Enterobacter* species	2
			*Pseudomonas* species	1
Bobic et al, 1993^111^	Tibial plateau	Plate and screws	*S aureus*	1
Lee et al, 2007^113^	Tibial plateau	Plate and screws	*S aureus *	1
Marsh et al, 1995^74^	Tibial plateau	Plate and screws	*S aureus *	2
Zhu et al, 2017^84^	Tibial plateau	Plate and screws	MRSA	3
			*S aureus*	2
Ma et al, 2018^83^	Tibial plateau	Plate and screws	*S aureus*	9
			*S epidermidis *	4
			Multiple genus and species	3
			*Pseudomonas aeruginosa *	1
Singh et al, 2015^125^	Tibial plateau or proximal tibia	Plate and screws	MRSA	4
			*S aureus*	6
			*S epidermidis*	1
			*E coli*	1
			*Enterobacter aerogenes*	1
			No culture performed	3
Torchia et al, 1996^36^	Patella	Tension band or other	*Streptococcus* species	2
			*Pseudomonas* species	2
			*S aureus*	1
			*Enterobacter* species	1
			*Peptococcus magnus*	1

Abbreviation: MRSA, methicillin-resistant *Staphylococcus aureus.*

### Subset Analysis

**Figure 3.**
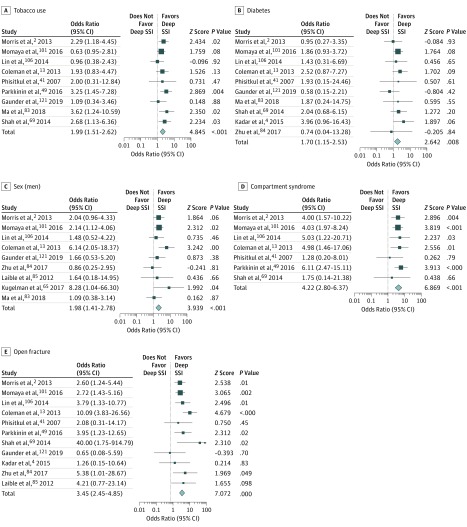
Subset Analyses of Risk Factors Associated With Deep Surgical Site Infection (SSI) Size of boxes indicates proportional weight of each trial. Diamonds indicate point estimates and 95% CIs of the combined result.

We analyzed factors associated with deep periarticular knee infection via subset analysis (Table 2). Our analysis found a statistically significant prevalence of periarticular knee infections in smokers (OR, 1.99; 95% CI, 1.51-2.62; *P* < .001) (Figure 3A). Additionally, patients with diabetes were also associated with an increased risk of developing deep periarticular knee SSIs (OR, 1.70; 95% CI, 1.15-2.53; *P* = .008) (Figure 3B). Men had a higher risk of developing deep periarticular knee infections than women (OR, 1.98; 95% CI, 1.41-2.78; *P* < .001;) (Figure 3C). Additionally, a statistically significant increased risk was found among patients with compartment syndrome compared with those without (OR, 4.22; 95% CI, 2.80-6.37; *P* < .001) (Figure 3D) and among patients with open fractures compared with those with closed fractures (OR, 3.45; 95% CI, 2.45-4.85; *P* < .001) (Figure 3E).

**Table 2.** Risk Factor Subset Analysis

Factor	Studies, No.	No. Infected/Total No. (%)	Odds Ratio (95% CI)	*P* Value	*I*^2^, %
Smoking status	9	246/2562 (9.6)			
Smoker		121/801 (15.1)	1.99 (1.51-2.62)	<.001	0
Nonsmoker		125/1761 (7.6)	1 [Reference]		
Diabetes status	10	237/2826 (8.4)			
With diabetes		35/244 (14.3)	1.70 (1.15-2.53)	.008	0
Without diabetes		202/2582 (7.8)	1 [Reference]		
Sex	9	206/2765 (7.5)			
Men		158/1790 (8.8)	1.98 (1.41-2.78)	<.001	14.51
Women		48/975 (4.9)	1 [Reference]		
Compartment syndrome status	7	193/1636 (11.8)			
With compartment syndrome		44/130 (33.8)	4.22 (2.80-6.37)	<.001	0
Without compartment syndrome		149/1506 (9.9)	1 [Reference]		
Fracture type	11	232/2225 (10.4)			
Open		61/253 (24.1)	3.45 (2.45-4.85)	<.001	16.71
Closed		171/1972 (8.7)	1 [Reference]		

## Discussion

Periarticular fractures around the knee are unique injuries, as they require the restoration of complex bone anatomy of the distal femur and proximal tibia as well as recovery of the surrounding soft-tissue envelope to promote return of knee function. Additionally, the soft-tissue anatomy around the knee is very intricate, including the extensor mechanism, knee capsule, meniscal cartilage, supporting ligaments, and tendinous attachments. These structures are often injured with the associated fractures and require repair or reconstruction to preserve knee function. Finally, the overlying soft-tissue envelope around the knee is thin and limited in the amount of mobilization or surgical insult it can tolerate. Overwhelming soft-tissue trauma or loss can lead to the need for soft-tissue reconstruction with rotational flaps or free tissue transfer.

Furthermore, periarticular knee fractures are often intraarticular, which makes them prone to developing traumatic arthritis. Anatomic reduction is necessary to help preserve the articular surface. Opening the joint is usually required to reconstruct an anatomic joint. If an SSI develops, deep infection into the joint can ensue. Our study found an overall prevalence of deep SSI of nearly 6%, and the prevalence of septic arthritis was 2.5%. Joint sepsis can further compromise knee function and overall outcomes. Additionally, septic arthritis may preclude salvage procedures, such as knee arthroplasty, which might be the only option to restore function to a badly damaged knee joint.

The type of injury to the soft-tissue envelope is a major determinant to the timing of surgical intervention of periarticular knee fractures. Open fractures require emergent incision and debridement of the soft tissue and often delayed definitive fixation of the skeletal tissues. Definitive skeletal stabilization can be performed only after the soft tissues are stabilized and tissue edema and swelling are resolving. If this process is rushed, the result is often catastrophic, as deep SSI will often ensue after wound breakdown or further loss of soft tissues that did not have adequate time to recover prior to further surgical trauma. This may be the most common reason rates of SSI are highest around the proximal tibia where the soft-tissue envelope is very thin.

In this study, a deep SSI rate of 5.7% and a superficial SSI rate of 3.4% were observed. Our results indicated that proximal tibia fractures were associated with the highest rate of deep SSI, observed in 6.4% of patients who underwent proximal tibia fracture operations; however, this rate was based on a smaller number of patients and was not statistically significant, as the CI overlapped with those for other fracture locations. Among the SSI incidents that included laboratory data, MRSA and methicillin-susceptible *S aureus* were the most commonly found bacteria. Surveillance data from the US Centers for Disease Control and Prevention and European Centre for Disease Control report MRSA prevalence at 1% and 5%, respectively. Considering that MRSA was the most common pathogen in our study and that this pathogen is increasing in prevalence, health care practitioners should revisit the use of specific and appropriate prophylactic antibiotics, especially in patients who exhibit known risk factors.

Subset analysis revealed that compared with the overall prevalence of SSI, smokers experienced a 3-fold greater SSI rate of 17.8%. Patients with compartment syndrome were associated with a severe predisposition to SSI, with a rate of 33.8%. This is consistent with a 2017 study by Shao et al^122^ that emphasized the association of compartment syndrome with the development of SSI. Open fractures were also associated with a greater risk of SSI, with patients with open fractures experiencing SSIs at a rate of 24.1%. This is a 3-fold increase from the 8.7% rate of SSI among patients with closed fractures. Additionally, patients with diabetes experienced SSI at a rate of 14.3% compared with 7.8% among patients who did not have diabetes. The results also revealed that men have nearly a 2-fold increased risk of SSI compared with women (8.8% vs 4.9%). Aside from open fractures and sex, these conditions illustrate the importance of a viable, healthy soft tissue envelope and adequate blood flow to the injured limb. The microvascular blood flow in patients with a smoking history, diabetes, or compartment syndrome is likely impaired, putting these individuals at a higher risk of a deep SSI owing to a decreased ability to deliver antibiotics or adequate white blood cells to the region to fight infection. Open fractures may have a higher risk of infection owing to a direct bacterial inoculum at the time of injury which may be accompanied by surrounding devitalized tissue injury that is not debrided in a timely and adequate manner.

The prevalence of septic arthritis in our study was based on a small number of studies and patients, which may be prone to bias or underclassification of septic arthritis. This is because not every study delineated whether the deep SSIs reported were within the joint. Thus, we propose that future studies should more clearly delineate within their samples the incidence of deep SSIs that are or lead to septic arthritis.

### Limitations

Our study had limitations. Many studies that have been conducted do not include the incidence of infection, septic arthritis, responsible microorganism, or the prophylactic strategies used to reduce SSI rates. Best practices to reduce infectious adverse outcomes for perioperative management at the time of periarticular fracture cannot be made on the basis of this study but should be the focus of future interventional studies. Moreover, other important parameters that could have an effect on the development of an SSI, such as type of incision, (minimally invasive vs open approach), type of fracture (simple vs complex), length of surgery, type of injury (crush injury vs a fall), method of reduction used (open vs closed), were not included in the final analysis, as the information on these factors provided in the studies was limited. Although we created our search to be as inclusive as possible, it is likely that relevant studies were not returned by our search.

A further limitation of this study is the shortage of randomized control trials and a low mean CMS score, which can be attributed to the high percentage of retrospective studies used. In the authors’ opinion, this finding increases the value of this work. Because this is by far the most comprehensive systematic review taken regarding periarticular knee fractures and SSI rate to our knowledge, our sample of studies likely represents the body of orthopedic traumatology research as a whole. Furthermore, our study found that studies with higher methodological quality had a statistically significant decrease in the prevalence of deep SSI. This suggests that if orthopedic surgeons design higher-quality studies, patient outcomes may be improved compared with patients included in lower quality studies. Recent studies^127,128,129,130^ further reinforce this sentiment that the room for improvement in orthopedic research quality is vast. In addition, poor quality studies are not necessarily inaccurate ones.

There was a high degree of asymmetry and heterogeneity in our sample of studies. First, the quality of many of the studies included in our review was poor, and retrospective studies were likely pooled in our assessment, adding to the asymmetry. Next, because our evaluation was comprised of studies in multiple fracture sites, from multiple countries, and of varying sizes, this could also contribute to the asymmetry of our funnel plot. It is also possible for differences in underlying risk, effect measures, and intensity of intervention to result in an asymmetric funnel plot. It must be acknowledged that some degree of publication bias is possible when funnel plots are asymmetric, but our large sample size renders this unlikely.

Greater efforts must be taken by those conducting orthopedic research to improve study design and limit methodological bias. Authors in orthopedic traumatology should strive to conduct higher-quality research, such as randomized clinical trials and case-control or cohort studies. Greater knowledge of quality assessment scores, such as those conducted by the Cochrane Collaboration, and the CMS may help researchers prospectively structure their studies in a manner that will be methodologically robust. Our hope is that by bringing attention to the poor quality of studies comprising a large sample of orthopedic traumatology research, future studies will pay greater attention to improving quality and reducing bias. By doing so, future systematic reviews and meta-analyses in orthopedic traumatology may not have this same limitation.

## Conclusion

In this meta-analysis, the proximal tibia was associated with the highest risk of developing a deep SSI, although this association may be owing to a smaller number of studies on the area. Furthermore, deep SSIs appeared more commonly than superficial SSIs; however, we recognize this could be owing to underestimation of superficial SSIs in the data set. Therefore, surgeons managing periarticular knee fractures must remain vigilant when wounds are not pristine. Septic arthritis occurred at a rate of 2.5% in studies reporting this statistic. Risk factors, such as open fractures, diabetes, smoking, and, most importantly, compartment syndrome, should alert the treating surgeon to an increased risk. Further work is needed to mitigate the association of these conditions with SSI risk in periarticular knee fractures. Given that these risk factors are not easily influenced, they do not outline a clear method to reduce SSI but instead present conditions wherein heightened awareness from a surgeon could potentially decrease the occurrence of SSI. Future studies are needed to determine the timing of internal fixation for periarticular knee fractures in patients with compartment syndrome (eg, waiting until the soft-tissue envelope has healed), the use of local and new systemic antibiotics in open fracture management (eg, adding prophylaxis for MRSA in certain populations and clinical scenarios), the association of diabetes status with SSI risk (eg, delaying treatment until control of the blood glucose has been obtained), and the association of smoking cessation with risk of deep SSI. In addition, if indicated, evaluating the management of concomitant peripheral vascular disease associated with either diabetes or smoking could also be beneficial. Finally, researchers conducting future studies that focus on periarticular deep SSI rates should more thoroughly report the prevalence of infection within the joint.

## Notes

Supplement. **eTable.** Included Study Characteristics **eReferences.**
