# Error in Abstract and Methods

**DOI:** 10.1001/jamanetworkopen.2019.13513

**Published:** 2019-10-02

In the Original Investigation titled “Prevalence of Deep Surgical Site Infection After Repair of Periarticular Knee Fractures: A Systematic Review and Meta-analysis,”^1^ published August 23, 2019, the Data Extraction and Synthesis section of the Abstract and the Study Identification subsection of the Methods section should state that data analyses were conducted in October 2018. This article has been corrected.
