# Supplementary material for: Prevalence of Deep Surgical Site Infection After Repair of Periarticular Knee Fractures: A Systematic Review and Meta-analysis
Source: JAMA Netw Open. 2019 Aug 23;2(8):e199951. doi: 10.1001/jamanetworkopen.2019.9951 (PMC6714463; doi:10.1001/jamanetworkopen.2019.9951)
Supplement: Supplement. — eTable. Included Study Characteristics eReferences. [file jamanetwopen-2-e199951-s001.pdf]

Supplementary Online Content

Norris GR, Checketts JX, Scott JT, Vassar M, Norris BL, Giannoudis PV. Prevalence of deep surgical site infection after repair of periarticular knee fractures: a systematic review and meta-analysis. *JAMA Netw Open*. 2019;2(8):e199951. doi:10.1001/jamanetworkopen.2019.9951

eTable. Included Study Characteristics
eReferences.

This supplementary material has been provided by the authors to give readers additional information about their work.

© 2019 Norris GR et al. *JAMA Network Open.*

eTable. Included Study Characteristics

Author	Type of Study	Total Sample Size	Incidence of Deep Infection	Incidence of Septic Arthritis	Fracture Location	Type of Hardware	Time to Infection
Steinberg, 2017[21]	Retrospective Chart Review	32	1	Not Reported	Distal Femur	Plate & Screws	Not Reported
Canadian Orthopaedic Trauma Society, 2016[22]	Randomized Controlled Trial	52	2	Not Reported	Distal Femur	Plate & Screws	Not Reported
Padha, 2016[23]	Prospective Trial	50	1	Not Reported	Distal Femur	Plate & Screws	Not Reported
Virk, 2016[24]	Prospective Trial	25	0	Not Reported	Distal Femur	Plate & Screws	Not Reported
Gill, 2017[25]	Randomized Controlled Trial	42	2	Not Reported	Distal Femur	Retrograde Nail, Plate & Screws	Not Reported
Kim, 2009[26]	Retrospective Chart Review	31	0	Not Reported	Distal Femur	Retrograde Nail	Not Reported
Kolb, 2008[27]	Retrospective Chart Review	31	1	Not Reported	Distal Femur	Plate & Screws	Not Reported
Rademakers, 2004[28]	Retrospective Chart Review	67	7	Not Reported	Distal Femur	Plate & Screws	Not Reported
Southeast Fracture Consortium, 2016[29]	Retrospective Chart Review	339	31	Not Reported	Distal Femur	Plate & Screws	Not Reported
Handolin, 2004[30]	Retrospective Chart Review	44	0	0	Distal Femur	Retrograde Nail	Not Reported
Gao, 2013[31]	Retrospective Chart Review	36	1	Not Reported	Distal Femur	Retrograde Nail, Plate & Screws	5 months
Schandelmaier, 1999[32]	Prospective Trial	29	3	Not Reported	Distal Femur	Plate & Screws	16 weeks, 7 months
Kayali, 2007[33]	Prospective Trial	27	2	Not Reported	Distal Femur	Plate & Screws	Not Reported
Kolb, 2009[34]	Retrospective Chart Review	41	2	Not Reported	Distal Femur	Plate & Screws	Not Reported

© 2019 Norris GR et al. *JAMA Network Open.*

Kregor, 2004[35]	Retrospective Chart Review	103	3	Not Reported	Distal Femur	Plate & Screws	Within 4 weeks
Schutz, 2005[36]	Prospective Trial	62	2	Not Reported	Distal Femur	Plate & Screws	Not Reported
Ran, 2013[37]	Retrospective Chart Review	28	1	Not Reported	Distal Femur, Proximal Tibia	Plate & Screws	Not Reported
Parekh, 2008[38]	Retrospective Chart Review	47	8	Not Reported	Distal Femur, Proximal Tibia	Other	Not Reported
Liu, 2009[1]	Retrospective Chart Review	189	1	Not Reported	Distal Femur, Tibial Plateau, Proximal Tibia	Plate & Screws	Not Reported
Kadar, 2016[39]	Retrospective Chart Review	60	6	1	Patella	Other	Not Reported
Hoshino, 2013[5]	Retrospective Chart Review	448	16	Not Reported	Patella	Tension Band	Not Reported
Bostman, 1981[40]	Retrospective Chart Review	64	0	0	Patella	Tension Band	Not Reported
Smith, 1997[41]	Retrospective Chart Review	51	0	Not Reported	Patella	Tension Band	Not Reported
Hsu, 2017[42]	Retrospective Chart Review	170	4	Not Reported	Patella	Tension Band	Not Reported
Torchia, 1996[43]	Retrospective Chart Review	47	6	Not Reported	Patella	Tension Band, Other	Not Reported
Anand, 2007[44]	Retrospective Chart Review	32	2	Not Reported	Patella	Tension Band, Other	Not Reported
Wu, 2001[45]	Prospective Trial	68	0	Not Reported	Patella	Tension Band	Not Reported
Kadar, 2015[46]	Retrospective Chart Review	188	11	Not Reported	Patella	Tension Band, Other	Not Reported
Uvaraj, 2007[47]	Retrospective Chart Review	22	2	Not Reported	Patella	Tension Band	Not Reported

© 2019 Norris GR et al. *JAMA Network Open.*

Wu, 2015[48]	Retrospective Chart Review	20	0	Not Reported	Proximal Tibia	Plate & Screws	Not Reported
Phisitkul, 2007[49]	Retrospective Chart Review	43	8	Not Reported	Proximal Tibia	Plate & Screws	3.4 months (Range 7-315 days)
Oh, 2006[50]	Retrospective Chart Review	211	0	Not Reported	Proximal Tibia	Plate & Screws	Not Reported
Zhang, 2015[51]	Prospective Trial	35	0	Not Reported	Proximal Tibia	Plate & Screws	Not Reported
Singh, 2015a[52]	Prospective Trial	20	0	Not Reported	Proximal Tibia	Other	Not Reported
Luo, 2006[53]	Prospective Trial	42	0	Not Reported	Proximal Tibia	Plate & Screws	Not Reported
Jockel, 2013[54]	Prospective Trial	86	2	Not Reported	Proximal Tibia	Plate & Screws	Not Reported
Kim, 2012[55]	Retrospective Chart Review	30	5	Not Reported	Proximal Tibia	Plate & Screws	Not Reported
Nikolaou, 2011[56]	Prospective Trial	55	2	2	Proximal Tibia	Plate & Screws	Not Reported
Parkkinin, 2016[57]	Retrospective Chart Review	170	34	Not Reported	Proximal Tibia	Plate & Screws	Not Reported
Egol, 2005[58]	Prospective Trial	57	3	1	Proximal Tibia	Plate & Screws, Other	3 months
Boldin, 2006[59]	Prospective Trial	26	0	Not Reported	Proximal Tibia	Plate & Screws	Not Reported
Cole, 2004[60]	Prospective Trial	77	2	Not Reported	Proximal Tibia	Plate & Screws	Not Reported
Jiang, 2008[61]	Prospective Trial	84	5	Not Reported	Tibial Plateau	Plate & Screws	Not Reported
Yao, 2015[62]	Randomized Controlled Trial	85	0	Not Reported	Tibial Plateau	Plate & Screws	Not Reported
Gross, 2017[63]	Retrospective Chart Review	40	0	0	Tibial Plateau	Plate & Screws	Not Reported

© 2019 Norris GR et al. *JAMA Network Open.*

Basques, 2015[64]	Retrospective Chart Review	519	6	5	Tibial Plateau	Plate & Screws	16.1 days (+/- 8.9)
Yu, 2013[65]	Retrospective Chart Review	112	1	Not Reported	Tibial Plateau	Plate & Screws	Not Reported
Abghari, 2016[66]	Prospective Trial	77	1	Not Reported	Tibial Plateau	Plate & Screws	Not Reported
Chan, 2003[67]	Prospective Trial	18	0	Not Reported	Tibial Plateau	Plate & Screws	Not Reported
Ali, 2001[68]	Prospective Trial	20	0	0	Tibial Plateau	Plate & Screws	Not Reported
Stamer, 1994[69]	Retrospective Chart Review	23	3	Not Reported	Tibial Plateau	Plate & Screws	Not Reported
Ferreira, 2014[70]	Retrospective Chart Review	46	0	0	Tibial Plateau	Plate & Screws	Not Reported
Sun, 2015[71]	Prospective Trial	41	0	Not Reported	Tibial Plateau	Plate & Screws	Not Reported
Lee, 2014[72]	Retrospective Chart Review	45	3	Not Reported	Tibial Plateau	Plate & Screws	Not Reported
Chan, 2012[73]	Retrospective Chart Review	59	2	1	Tibial Plateau	Plate & Screws	Not Reported
Kugelman, 2017[74]	Prospective Trial	279	10	Not Reported	Tibial Plateau	Plate & Screws	Not Reported
Barei, 2004[75]	Retrospective Chart Review	83	7	3	Tibial Plateau	Plate & Screws	5 w/i 3 weeks, 1 at 8 weeks, 1 at 7 months
Khatri, 2016[76]	Retrospective Chart Review	62	5	Not Reported	Tibial Plateau	Plate & Screws	Not Reported
Ruffolo, 2015[77]	Retrospective Chart Review	140	33	7	Tibial Plateau	Plate & Screws	28 occured at less than 6 months, 5 occured at 6-46 months

© 2019 Norris GR et al. *JAMA Network Open.*

Shah, 2014[78]	Retrospective Chart Review	187	25	Not Reported	Tibial Plateau	Plate & Screws	Not Reported
Haller, 2016[79]	Retrospective Chart Review	159	11	Not Reported	Tibial Plateau	Plate & Screws	Not Reported
Ozkaya, 2015[80]	Retrospective Chart Review	108	1	Not Reported	Tibial Plateau	Plate & Screws	4 months
Shah, 2007[81]	Retrospective Chart Review	29	4	Not Reported	Tibial Plateau	Plate & Screws	16 days - 3.5 months
Chen, 2018[82]	Prospective Trial	240	0	Not Reported	Tibial Plateau	Plate & Screws	Not Reported
Marsh, 1995[83]	Retrospective Chart Review	21	2	2	Tibial Plateau	Plate & Screws	3 weeks
Tao, 2017[84]	Retrospective Chart Review	34	0	Not Reported	Tibial Plateau	Plate & Screws	Not Reported
Stannard, 2003[85]	Prospective Trial	35	2	Not Reported	Tibial Plateau	Plate & Screws	Not Reported
Berg, 2017[86]	Retrospective Chart Review	148	6	Not Reported	Tibial Plateau	Plate & Screws	Not Reported
Prasad, 2013[87]	Retrospective Chart Review	40	0	Not Reported	Tibial Plateau	Plate & Screws	Not Reported
Yao, 2014[88]	Retrospective Chart Review	74	0	Not Reported	Tibial Plateau	Plate & Screws	Not Reported
Barei, 2006[89]	Retrospective Chart Review	41	2	2	Tibial Plateau	Plate & Screws	Not Reported
Babis, 2011[90]	Retrospective Chart Review	33	1	1	Tibial Plateau	Plate & Screws	Not Reported
Keightley, 2015[91]	Prospective Trial	105	0	Not Reported	Tibial Plateau	Plate & Screws	Not Reported
Ma, 2018[92]	Retrospective Chart Review	676	17	Not Reported	Tibial Plateau	Plate & Screws	9 days
Zhu, 2017[93]	Prospective Trial	235	5	Not Reported	Tibial Plateau	Plate & Screws	median of 6 days
Laible, 2012[94]	Retrospective Chart Review	79	6	Not Reported	Tibial Plateau	Plate & Screws	Not Reported

© 2019 Norris GR et al. *JAMA Network Open.*

Meena, 2015[95]	Randomized Controlled Trial	44	1	Not Reported	Tibial Plateau	Plate & Screws, Suprapatellar Nail	Not Reported
Borade, 2017[96]	Retrospective Chart Review	81	4	Not Reported	Tibial Plateau	Plate & Screws	Not Reported
Maroto, 2013[97]	Retrospective Chart Review	85	11	Not Reported	Tibial Plateau	Plate & Screws	Not Reported
Jansen, 2013[98]	Prospective Trial	22	2	Not Reported	Tibial Plateau	Plate & Screws	Not Reported
Van Dreumel, 2015[99]	Retrospective Chart Review	71	1	Not Reported	Tibial Plateau	Plate & Screws	Not Reported
Giannetti, 2017[100]	Prospective Trial	40	0	Not Reported	Tibial Plateau	Plate & Screws	Not Reported
Ariffin, 2011[101]	Prospective Trial	31	1	1	Tibial Plateau	Other	2 weeks
Zhai, 2014[102]	Retrospective Chart Review	29	1	Not Reported	Tibial Plateau	Plate & Screws	Not Reported
Rademakers, 2007[103]	Retrospective Chart Review	202	11	Not Reported	Tibial Plateau	Plate & Screws	Within 2 weeks
Su, 2004[104]	Retrospective Chart Review	38	1	Not Reported	Tibial Plateau	Plate & Screws	Not Reported
Giordano, 2017[105]	Retrospective Chart Review	30	1	Not Reported	Tibial Plateau	Plate & Screws	Not Reported
Ali, 2013[106]	Retrospective Chart Review	25	1	Not Reported	Tibial Plateau	Plate & Screws	Not Reported
Colman, 2013[107]	Retrospective Chart Review	309	24	Not Reported	Tibial Plateau	Plate & Screws	Not Reported
Urruela, 2013[108]	Prospective Trial	96	2	Not Reported	Tibial Plateau	Plate & Screws	Not Reported
Conserva, 2015[109]	Retrospective Chart Review	79	6	Not Reported	Tibial Plateau	Plate & Screws	Not Reported
Momaya, 2016[110]	Retrospective Chart Review	532	59	Not Reported	Tibial Plateau	Plate & Screws	Not Reported

© 2019 Norris GR et al. *JAMA Network Open.*

Morris, 2013[2]	Retrospective Chart Review	302	43	Not Reported	Tibial Plateau	Plate & Screws	Not Reported
Bertrand, 2017[111]	Retrospective Chart Review	93	10	Not Reported	Tibial Plateau	Plate & Screws, Other	Not Reported
Gosling, 2005[112]	Prospective Trial	62	1	Not Reported	Tibial Plateau	Plate & Screws	Not Reported
Kataria, 2007[113]	Retrospective Case Series	22	0	Not Reported	Tibial Plateau	Other	Not Reported
Bagherifard, 2016[114]	Prospective Trial	32	1	Not Reported	Tibial Plateau	Plate & Screws	Not Reported
Lin, 2014[115]	Retrospective Chart Review	256	16	Not Reported	Tibial Plateau	Plate & Screws	Within 4 weeks
Dubina, 2017[116]	Retrospective Chart Review	703	69	Not Reported	Tibial Plateau	Plate & Screws	Not Reported
Faldini, 2005[117]	Retrospective Chart Review	32	0	Not Reported	Tibial Plateau	Other	Not Reported
Stannard, 2004[118]	Prospective Trial	39	0	Not Reported	Tibial Plateau	Plate & Screws	Not Reported
Ahearn, 2014[119]	Retrospective Chart Review	55	1	Not Reported	Tibial Plateau	Plate & Screws	Not Reported
Bobic, 1993[120]	Retrospective Case Series	31	1	1	Tibial Plateau	Plate & Screws	Not Reported
Russell, 2009[121]	Retrospective Chart Review	60	0	Not Reported	Tibial Plateau	Plate & Screws	Not Reported
Lee, 2007[122]	Retrospective Chart Review	35	2	Not Reported	Tibial Plateau	Plate & Screws	Not Reported
Manidakis, 2010[123]	Retrospective Chart Review	125	12	8	Tibial Plateau	Plate & Screws	Not Reported
Biggi, 2010[124]	Retrospective Chart Review	58	1	Not Reported	Tibial Plateau	Plate & Screws	Not Reported
Zura, 2010[125]	Retrospective Chart Review	30	5	Not Reported	Tibial Plateau	Plate & Screws	7 weeks

© 2019 Norris GR et al. *JAMA Network Open.*

Tang, 2012[126]	Retrospective Chart Review	42	0	Not Reported	Tibial Plateau	Plate & Screws	Not Reported
Kumar, 2000[127]	Retrospective Chart Review	57	4	2	Tibial Plateau	Other	Not Reported
Egol, 2004[128]	Retrospective Chart Review	74	2	1	Tibial Plateau	Plate & Screws	Not Reported
Dendrinos, 1996[129]	Prospective Trial	24	0	Not Reported	Tibial Plateau	Plate & Screws	Not Reported
Gaunder, 2018[130]	Retrospective Chart Review	102	12	Not Reported	Tibial Plateau	Plate & Screws	Not Reported
Yoon, 2015[131]	Retrospective Chart Review	27	2	Not Reported	Tibial Plateau, Proximal Tibia		Not Reported
Iliopoulos, 2017[132]	Retrospective Chart Review	70	0	Not Reported	Tibial Plateau, Proximal Tibia	Plate & Screws	Not Reported
Singh, 2015b[133]	Retrospective Chart Review	93	15	Not Reported	Tibial Plateau, Proximal Tibia	Plate & Screws	Not Reported
Lovisseti, 2018[134]	Retrospective Chart Review	20	1	Not Reported	Tibial Plateau, Proximal Tibia	Other	Not Reported

© 2019 Norris GR et al. *JAMA Network Open.*

eReferences.

1. Liu F, Tao R, Cao Y, et al. The role of LISS (less invasive stabilisation system) in the treatment of peri-knee fractures. *Injury*. 2009;40(11):1187-1194.

2. Morris BJ, Unger RZ, Archer KR, Mathis SL, Perdue AM, Obremskey WT. Risk factors of infection after ORIF of bicondylar tibial plateau fractures. *J Orthop Trauma*. 2013;27(9):e196-e200.

3. Rodriguez EK, Boulton C, Weaver MJ, et al. Predictive factors of distal femoral fracture nonunion after lateral locked plating: a retrospective multicenter case-control study of 283 fractures. *Injury*. 2014;45(3):554-559.

4. A. K, H. S, Y. G, E. K, E.I. S. Predictors for nonunion, reoperation and infection after surgical fixation of patellar fracture. *J Ortop Sci*. 2015;20(1):168-173.

5. Hoshino CM, Tran W, Tiberi JV, et al. Complications following tension-band fixation of patellar fractures with cannulated screws compared with Kirschner wires. *J Bone Joint Surg Am*. 2013;95(7):653-659.

6. LeBrun CT, Langford JR, Sagi HC. Functional outcomes after operatively treated patella fractures. *J Orthop Trauma*. 2012;26(7):422-426.

7. Petrie J, Sassoon A, Langford J. Complications of patellar fracture repair: treatment and results. *J Knee Surg*. 2013;26(5):309-312.

8. Henkelmann R, Frosch K-H, Glaab R, et al. Infection following fractures of the proximal tibia - a systematic review of incidence and outcome. *BMC Musculoskelet Disord*. 2017;18(1):481.

9. Heppert V, Rheinwalt K, Winkler H, Wentzensen A. Infection of the proximal tibia after fractures—An avoidable complication. *Eur J Orthop Surg Traumatol*. 1997;7(3):195-198.

10. B.a. B, M.l. W, D.d. B, N.s. G, J.n. G. Adverse events, length of stay, and readmission following surgery for tibial plateau fractures. *J Orthop Trauma*. January 2014.

11. Kirkland KB, Briggs JP, Trivette SL, Wilkinson WE, Sexton DJ. The impact of surgical-site infections in the 1990s: attributable mortality, excess length of hospitalization, and extra costs. *Infect Control Hosp Epidemiol*. 1999;20(11):725-730.

12. Moher D, Liberati A, Tetzlaff J, Altman DG, PRISMA Group. Preferred reporting items for systematic reviews and meta-analyses: the PRISMA statement. *Ann Intern Med*. 2009;151(4):264-269, W64.

13. Liberati A, Altman DG, Tetzlaff J, et al. The PRISMA statement for reporting systematic reviews and meta-analyses of studies that evaluate healthcare interventions: explanation and elaboration. *BMJ*. 2009;339:b2700.

14. Saleeb H, Tosounidis T, Papakostidis C, Giannoudis PV. Incidence of deep infection, union and malunion for open diaphyseal femoral shaft fractures treated with IM nailing: A systematic review. *Surgeon*. August 2018. doi:10.1016/j.surge.2018.08.003

© 2019 Norris GR et al. *JAMA Network Open.*

15. Coleman BD, Khan KM, Maffulli N. Studies of surgical outcome after patellar tendinopathy: clinical significance of methodological deficiencies and guidelines for future studies. *J Med*. 2000. https://onlinelibrary.wiley.com/doi/abs/10.1034/j.1600-0838.2000.010001002.x.

16. Shao J, Chang H, Zhu Y, et al. Incidence and risk factors for surgical site infection after open reduction and internal fixation of tibial plateau fracture: A systematic review and meta-analysis. *Int J Surg*. 2017;41:176-182.

17. Scott J, Checketts JX, Horn JG, Cooper C, Vassar M. "Knee osteoarthritis and current research for evidence-are we on the right way?" *Int Orthop*. 2018;42(9):2105-2112.

18. Checketts JX, Scott J, Gordon J, et al. An Evaluation of the Rotator Cuff Repair Research Pipeline. *Orthop J Sports Med*. 2018;6(11):2325967118805731.

19. Checketts JX, Sims MT, Detweiler B, Middlemist K, Jones J, Vassar M. An Evaluation of Reporting Guidelines and Clinical Trial Registry Requirements Among Orthopaedic Surgery Journals. *J Bone Joint Surg Am*. 2018;100(3):e15.

20. Checketts JX, Scott JT, Meyer C, Horn J, Jones J, Vassar M. The Robustness of Trials That Guide Evidence-Based Orthopaedic Surgery. *J Bone Joint Surg Am*. 2018;100(12):e85.

21. Steinberg EL, Elis J, Steinberg Y, Salai M, Ben-Tov T. A double-plating approach to distal femur fracture: A clinical study. *Injury*. 2017;48(10):2260-2265.

22. Canadian Orthopaedic Trauma Society. Are Locking Constructs in Distal Femoral Fractures Always Best? A Prospective Multicenter Randomized Controlled Trial Comparing the Less Invasive Stabilization System With the Minimally Invasive Dynamic Condylar Screw System. *J Orthop Trauma*. 2016;30(1):e1-e6.

23. Kanav Padha, Sandeep Singh, Abdul Ghani, Harish Dang. Distal Femur Fractures and its Treatment with Distal Femur Locking Plate. *JK Science*. 2016;18(2). http://jkscience.org/archives/volume182/5-Original%20Article.pdf.

24. Virk JS, Garg SK, Gupta P, Jangira V, Singh J, Rana S. Distal Femur Locking Plate: The Answer to All Distal Femoral Fractures. *J Clin Diagn Res*. 2016;10(10):RC01-RC05.

25. Gill S, Mittal A, Raj M, Singh P, Singh J, Kumar S. Extra Articular Supracondylar Femur Fractures Managed with Locked Distal Femoral Plate or Supracondylar Nailing: A Comparative Outcome Study. *J Clin Diagn Res*. 2017;11(5):RC19-RC23.

26. Kim J-W, Oh C-W, Kyung H-S, Min W-K, Yoon S-H. Factors affecting the results of distal femoral fractures treated by retrograde intramedullary nailing. *Zhongguo Xiu Fu Chong Jian Wai Ke Za Zhi*. 2009;23(11):1311-1315.

27. Kolb W, Guhlmann H, Windisch C, Marx F, Kolb K, Koller H. Fixation of distal femoral fractures with the Less Invasive Stabilization System: a minimally invasive treatment with locked fixed-angle screws. *J Trauma*. 2008;65(6):1425-1434.

28. Rademakers MV, Kerkhoffs GMMJ, Sierevelt IN, Raaymakers ELFB, Marti RK. Intra-Articular Fractures of the Distal Femur: A Long-Term Follow-up Study of Surgically Treated Patients. *J Orthop Trauma*. 2004;18(4):213-219.

© 2019 Norris GR et al. *JAMA Network Open.*

29. Southeast Fracture Consortium. LCP Versus LISS in the Treatment of Open and Closed Distal Femur Fractures: Does it Make a Difference? *J Orthop Trauma*. 30(6):e212-e216.

30. Handolin L, Pajarinen J, Lindahl J, Hirvensalo E. Retrograde intramedullary nailing in distal femoral fractures—results in a series of 46 consecutive operations. *Injury*. 2004;35(5):517-522.

31. Gao K, Gao W, Huang J, et al. Retrograde nailing versus locked plating of extra-articular distal femoral fractures: comparison of 36 cases. *Med Princ Pract*. 2013;22(2):161-166.

32. Schandelmaier P, Krettek C, Miclau T, Stephan C, Könemann B, Tscherne H. Stabilization of Distal Femoral Fractures Using the LISS. *Techniques in Orthopaedics*. 14(3):230-246.

33. Kayali C, Agus H, Turgut A. Successful results of minimally invasive surgery for comminuted supracondylar femoral fractures with LISS: comparative study of multiply injured and isolated femoral fractures. *J Orthop Sci*. 2007;12(5):458-465.

34. Kolb K, Grützner P, Koller H, Windisch C, Marx F, Kolb W. The condylar plate for treatment of distal femoral fractures: a long-term follow-up study. *Injury*. 2009;40(4):440-448.

35. Kregor PJ, Stannard JA, Zlowodzki M, Cole PA. Treatment of distal femur fractures using the less invasive stabilization system: surgical experience and early clinical results in 103 fractures. *J Orthop Trauma*. 2004;18(8):509-520.

36. Schütz M, Müller M, Regazzoni P, et al. Use of the less invasive stabilization system (LISS) in patients with distal femoral (AO33) fractures: a prospective multicenter study. *Arch Orthop Trauma Surg*. 2005;125(2):102-108.

37. Ran T, Hua X, Zhenyu Z, et al. Floating knee: a modified Fraser's classification and the results of a series of 28 cases. *Injury*. 2013;44(8):1033-1042.

38. Parekh AA, Smith WR, Silva S, et al. Treatment of distal femur and proximal tibia fractures with external fixation followed by planned conversion to internal fixation. *J Trauma*. 2008;64(3):736-739.

39. Kadar A, Sherman H, Drexler M, Katz E, Steinberg EL. Anchor suture fixation of distal pole fractures of patella: twenty seven cases and comparison to partial patellectomy. *Int Orthop*. 2016;40(1):149-154.

40. Böstman O, Kiviluoto O, Nirhamo J. Comminuted displaced fractures of the patella. *Injury*. 1981;13(3):196-202.

41. Smith ST, Cramer KE, Karges DE, Watson JT, Moed BR. Early complications in the operative treatment of patella fractures. *J Orthop Trauma*. 1997;11(3):183-187.

42. Hsu K-L, Chang W-L, Yang C-Y, Yeh M-L, Chang C-W. Factors affecting the outcomes of modified tension band wiring techniques in transverse patellar fractures. *Injury*. 2017;48(12):2800-2806.

43. Torchia ME, Lewallen DG. Open fractures of the patella. *J Orthop Trauma*. 1996;10(6):403-409.

44. Anand S, Hahnel JCR, Giannoudis PV. Open patellar fractures: high energy injuries with a

poor outcome? *Injury*. 2008;39(4):480-484.

45. Wu CC, Tai CL, Chen WJ. Patellar tension band wiring: a revised technique. *Arch Orthop Trauma Surg*. 2001;121(1):12-16.

46. Kadar A, Sherman H, Glazer Y, Katz E, Steinberg EL. Predictors for nonunion, reoperation and infection after surgical fixation of patellar fracture. *J Orthop Sci*. 2015;20(1):168-173.

47. Uvaraj NR, Mayil Vahanan N, Sivaseelam A, Mohd Sameer M, Basha IM. Surgical management of neglected fractures of the patella. *Injury*. 2007;38(8):979-983.

48. Wu D, Reng G, Shrivastava A, Yu Y, Zhang Y, Peng C. A useful surgical strategy for proximal tibial fractures (AO/OTA type 41-C) with diaphyseal involvement. *Int J Clin Exp Med*. 2015;8(8):13455-13463.

49. Phisitkul P, McKinley TO, Nepola JV, Marsh JL. Complications of locking plate fixation in complex proximal tibia injuries. *J Orthop Trauma*. 2007;21(2):83-91.

50. Oh C-W, Oh J-K, Kyung H-S, et al. Double plating of unstable proximal tibial fractures using minimally invasive percutaneous osteosynthesis technique. *Acta Orthop*. 2006;77(3):524-530.

51. Zhang J, Ebraheim N, Li M, et al. External fixation using femoral less invasive stabilization system plate in tibial proximal metaphyseal fracture. *Clin Orthop Surg*. 2015;7(1):8-14.

52. Singh H, Misra RK, Kaur M. Management of Proximal Tibia Fractures Using Wire Based Circular External Fixator. *J Clin Diagn Res*. 2015;9(9):RC01-RC04.

53. Luo C-F, Jiang R, Hu C-F, Zeng B-F. Medial double-plating for fracture dislocations involving the proximal tibia. *Knee*. 2006;13(5):389-394.

54. Jöckel JA, Erhardt J, Vincenti M, et al. Minimally invasive and open surgical treatment of proximal tibia fractures using a polyaxial locking plate system: a prospective multi-centre study. *Int Orthop*. 2013;37(4):701-708.

55. Kim J-W, Oh C-W, Jung W-J, Kim J-S. Minimally invasive plate osteosynthesis for open fractures of the proximal tibia. *Clin Orthop Surg*. 2012;4(4):313-320.

56. Nikolaou VS, Tan HB, Haidukewych G, Kanakaris N, Giannoudis PV. Proximal tibial fractures: early experience using polyaxial locking-plate technology. *Int Orthop*. 2011;35(8):1215-1221.

57. Parkkinen M, Madanat R, Lindahl J, Mäkinen TJ. Risk Factors for Deep Infection Following Plate Fixation of Proximal Tibial Fractures. *J Bone Joint Surg Am*. 2016;98(15):1292-1297.

58. Egol KA, Tejwani NC, Capla EL, Wolinsky PL, Koval KJ. Staged management of high-energy proximal tibia fractures (OTA types 41): the results of a prospective, standardized protocol. *J Orthop Trauma*. 2005;19(7):448-455; discussion 456.

59. Boldin C, Fankhauser F, Hofer HP, Szyszkowitz R. Three-year results of proximal tibia fractures treated with the LISS. *Clin Orthop Relat Res*. 2006;445:222-229.

60. Cole PA, Zlowodzki M, Kregor PJ. Treatment of proximal tibia fractures using the less

© 2019 Norris GR et al. *JAMA Network Open.*

invasive stabilization system: surgical experience and early clinical results in 77 fractures. *J Orthop Trauma*. 2004;18(8):528-535.

61. Jiang R, Luo C-F, Wang M-C, Yang T-Y, Zeng B-F. A comparative study of Less Invasive Stabilization System (LISS) fixation and two-incision double plating for the treatment of bicondylar tibial plateau fractures. *Knee*. 2008;15(2):139-143.

62. Yao Y, Lv H, Zan J, et al. A comparison of lateral fixation versus dual plating for simple bicondylar fractures. *Knee*. 2015;22(3):225-229.

63. Gross J-B, Gavanier B, Belleville R, Coudane H, Mainard D. Advantages of external hybrid fixators for treating Schatzker V-VI tibial plateau fractures: A retrospective study of 40 cases. *Orthop Traumatol Surg Res*. 2017;103(6):965-970.

64. Basques BA, Webb ML, Bohl DD, Golinvaux NS, Grauer JN. Adverse events, length of stay, and readmission after surgery for tibial plateau fractures. *J Orthop Trauma*. 2015;29(3):e121-e126.

65. Xu Y-Q, Li Q, Shen T-G, Su P-H, Zhu Y-Z. An efficacy analysis of surgical timing and procedures for high-energy complex tibial plateau fractures. *Orthop Surg*. 2013;5(3):188-195.

66. Abghari M, Marcano A, Davidovitch R, Konda SR, Egol KA. Are Locked Plates Needed for Split Depression Tibial Plateau Fractures? *J Knee Surg*. 2016;29(6):482-486.

67. Chan Y-S, Yuan L-J, Hung S-S, et al. Arthroscopic-assisted reduction with bilateral buttress plate fixation of complex tibial plateau fractures. *Arthroscopy*. 2003;19(9):974-984.

68. Ali AM, Yang L, Hashmi M, Saleh M. Bicondylar tibial plateau fractures managed with the Sheffield Hybrid Fixator: Biomechanical study and operative technique. *Injury*. 2001;32:86-91.

69. Stamer DT, Schenk R, Staggers B, Aurori K, Aurori B, Behrens FF. Bicondylar Tibial Plateau Fractures Treated with a Hybrid Ring External Fixator: A Preliminary Study. *J Orthop Trauma*. 8(6):455-461.

70. Ferreira N, Marais LC. Bicondylar tibial plateau fractures treated with fine-wire circular external fixation. *Strategies Trauma Limb Reconstr*. 2014;9(1):25-32.

71. Sun H, Zhai Q-L, Xu Y-F, Wang Y-K, Luo C-F, Zhang C-Q. Combined approaches for fixation of Schatzker type II tibial plateau fractures involving the posterolateral column: a prospective observational cohort study. *Arch Orthop Trauma Surg*. 2015;135(2):209-221.

72. Lee M-H, Hsu C-J, Lin K-C, Renn J-H. Comparison of outcome of unilateral locking plate and dual plating in the treatment of bicondylar tibial plateau fractures. *J Orthop Surg Res*. 2014;9:62.

73. Chan C, Keating J. Comparison of outcomes of operatively treated bicondylar tibial plateau fractures by external fixation and internal fixation. *Malays Orthop J*. 2012;6(1):7-12.

74. Kugelman D, Qatu A, Haglin J, Leucht P, Konda S, Egol K. Complications and unplanned outcomes following operative treatment of tibial plateau fractures. *Injury*. 2017;48(10):2221-2229.

© 2019 Norris GR et al. *JAMA Network Open.*

75. Barei DP, Nork SE, Mills WJ, Bradford Henley M, Benirschke SK. Complications Associated With Internal Fixation of High-Energy Bicondylar Tibial Plateau Fractures Utilizing a Two-Incision Technique. *J Orthop Trauma*. 18(10):649-657.

76. Khatri K, Sharma V, Goyal D, Farooque K. Complications in the management of closed high-energy proximal tibial plateau fractures. *Chin J Traumatol*. 2016;19(6):342-347.

77. Ruffolo MR, Gettys FK, Montijo HE, Seymour RB, Karunakar MA. Complications of high-energy bicondylar tibial plateau fractures treated with dual plating through 2 incisions. *J Orthop Trauma*. 2015;29(2):85-90.

78. Shah CM, Babb PE, McAndrew CM, et al. Definitive plates overlapping provisional external fixator pin sites: is the infection risk increased? *J Orthop Trauma*. 2014;28(9):518-522.

79. Haller JM, Holt D, Rothberg DL, Kubiak EN, Higgins TF. Does Early versus Delayed Spanning External Fixation Impact Complication Rates for High-energy Tibial Plateau and Plafond Fractures? *Clin Orthop Relat Res*. 2016;474(6):1436-1444.

80. Ozkaya U, Parmaksizoglu AS. Dual locked plating of unstable bicondylar tibial plateau fractures. *Injury*. 2015;46 Suppl 2:S9-S13.

81. Shah SN, Karunakar MA. Early wound complications after operative treatment of high energy tibial plateau fractures through two incisions. *Bull NYU Hosp Jt Dis*. 2007;65(2):115-119.

82. Chen G, Liang Y, Ruan J, Chen F, Wang H, Zhu G. Effect of modified dual plating and less invasive stabilization system on the treatment of complex fractures of tibial plateau. *Biomed Res*. 2018;28(22):10132-10136.

83. Marsh JL, Smith ST, Do TT. External fixation and limited internal fixation for complex fractures of the tibial plateau. *J Bone Joint Surg Am*. 1995;77(5):661-673.

84. Tao X, Chen N, Pan F, Cheng B. External fixation combined with delayed internal fixation in treatment of tibial plateau fractures with dislocation. *Medicine* . 2017;96(41):e8221.

85. Stannard JP, Wilson TC, Volgas DA, Alonso JE. Fracture Stabilization of Proximal Tibial Fractures with the Proximal Tibial LISS: Early Experience in Birmingham, Alabama (USA). *Injury*. 2003;34:S36-S42.

86. van den Berg J, Reul M, Nunes Cardozo M, et al. Functional outcome of intra-articular tibial plateau fractures: the impact of posterior column fractures. *Int Orthop*. 2017;41(9):1865-1873.

87. Prasad GT, Kumar TS, Kumar RK, Murthy GK, Sundaram N. Functional outcome of Schatzker type V and VI tibial plateau fractures treated with dual plates. *Indian J Orthop*. 2013;47(2):188-194.

88. Yao Y, Lv H, Zan J, Li J, Zhu N, Jing J. Functional outcomes of bicondylar tibial plateau fractures treated with dual buttress plates and risk factors: a case series. *Injury*. 2014;45(12):1980-1984.

89. Barei DP, Nork SE, Mills WJ, Coles CP, Henley MB, Benirschke SK. FUNCTIONAL OUTCOMES OF SEVERE BICONDYLAR TIBIAL PLATEAU FRACTURES TREATED

© 2019 Norris GR et al. *JAMA Network Open.*

WITH DUAL INCISIONS AND MEDIAL AND LATERAL PLATES. *The Journal of Bone and Joint Surgery-American Volume*. 2006;88(8):1713-1721.

90. Babis GC, Evangelopoulos DS, Kontovazenitis P, Nikolopoulos K, Soucacos PN. High energy tibial plateau fractures treated with hybrid external fixation. *J Orthop Surg Res*. 2011;6:35.

91. Keightley AJ, Nawaz SZ, Jacob JT, Unnithan A, Elliott DS, Khaleel A. Ilizarov management of Schatzker IV to VI fractures of the tibial plateau: 105 fractures at a mean follow-up of 7.8 years. *Bone Joint J*. 2015;97-B(12):1693-1697.

92. Ma Q, Aierxiding A, Wang G, Wang C, Yu L, Shen Z. Incidence and risk factors for deep surgical site infection after open reduction and internal fixation of closed tibial plateau fractures in adults. *Int Wound J*. 2018;15(2):237-242.

93. Zhu Y, Liu S, Zhang X, Chen W, Zhang Y. Incidence and risks for surgical site infection after adult tibial plateau fractures treated by ORIF: a prospective multicentre study. *Int Wound J*. 2017;14(6):982-988.

94. Laible C, Earl-Royal E, Davidovitch R, Walsh M, Egol KA. Infection after spanning external fixation for high-energy tibial plateau fractures: is pin site-plate overlap a problem? *J Orthop Trauma*. 2012;26(2):92-97.

95. Meena RC, Meena UK, Gupta GL, Gahlot N, Gaba S. Intramedullary nailing versus proximal plating in the management of closed extra-articular proximal tibial fracture: a randomized controlled trial. *J Orthop Traumatol*. 2015;16(3):203-208.

96. Borade A, Kempegowda H, Richard R, Graham J, Suk M, Horwitz DS. Is “Early Total Care” a Safe and Effective Alternative to “Staged Protocol” for the Treatment of Schatzker IV-VI Tibial Plateau Fractures in Patients Older Than 50 Years? *J Orthop Trauma*. 2017;31(12):e400-e406.

97. Maroto MD, Scolaro JA, Henley MB, Dunbar RP. Management and incidence of tibial tubercle fractures in bicondylar fractures of the tibial plateau. *Bone Joint J*. 2013;95-B(12):1697-1702.

98. Jansen H, Frey SP, Doht S, Fehske K, Meffert RH. Medium-term results after complex intra-articular fractures of the tibial plateau. *J Orthop Sci*. 2013;18(4):569-577.

99. van Dreumel RLM, van Wunnik BPW, Janssen L, Simons PCG, Janzing HMJ. Mid- to long-term functional outcome after open reduction and internal fixation of tibial plateau fractures. *Injury*. 2015;46(8):1608-1612.

100. Giannetti S, Bizzotto N, Stancati A, Santucci A. Minimally invasive fixation in tibial plateau fractures using an pre-operative and intra-operative real size 3D printing. *Injury*. 2017;48(3):784-788.

101. Ariffin HM, Mahdi NM, Rhani SA, Baharudin A, Shukur MH. Modified hybrid fixator for high-energy Schatzker V and VI tibial plateau fractures. *Strategies Trauma Limb Reconstr*. 2011;6(1):21-26.

102. Zhai Q, Hu C, Luo C. Multi-plate reconstruction for severe bicondylar tibial plateau

© 2019 Norris GR et al. *JAMA Network Open.*

fractures of young adults. *Int Orthop*. 2014;38(5):1031-1035.

103. Rademakers MV, Kerkhoffs GMMJ, Sierevelt IN, Raaymakers ELFB, Marti RK. Operative treatment of 109 tibial plateau fractures: five- to 27-year follow-up results. *J Orthop Trauma*. 2007;21(1):5-10.

104. Su EP, Westrich GH, Rana AJ, Kapoor K, Helfet DL. Operative Treatment of Tibial Plateau Fractures in Patients Older Than 55 Years. *Clin Orthop Relat Res*. 421:240-248.

105. Giordano V, do Amaral NP, Koch HA, Albuquerque RP e., de Souza FS, dos Santos Neto JF. Outcome evaluation of staged treatment for bicondylar tibial plateau fractures. *Injury*. 2017;48:S34-S40.

106. Ali AM. Outcomes of open bicondylar tibial plateau fractures treated with Ilizarov external fixator with or without minimal internal fixation. *Eur J Orthop Surg Traumatol*. 2013;23(3):349-355.

107. Colman M, Wright A, Gruen G, Siska P, Pape H-C, Tarkin I. Prolonged operative time increases infection rate in tibial plateau fractures. *Injury*. 2013;44(2):249-252.

108. Urruela AM, Davidovitch R, Karia R, Khurana S, Egol KA. Results following operative treatment of tibial plateau fractures. *J Knee Surg*. 2013;26(3):161-165.

109. Conserva V, Vicenti G, Allegretti G, et al. Retrospective review of tibial plateau fractures treated by two methods without staging. *Injury*. 2015;46(10):1951-1956.

110. Momaya AM, Hlavacek J, Etier B, et al. Risk factors for infection after operative fixation of Tibial plateau fractures. *Injury*. 2016;47(7):1501-1505.

111. Bertrand ML, Javier Pascual-López F, Guerado E. Severe tibial plateau fractures (Schatzker V–VI): open reduction and internal fixation versus hybrid external fixation. *Injury*. 2017;48:S81-S85.

112. Gosling T, Schandelmaier P, Muller M, Hankemeier S, Wagner M, Krettek C. Single Lateral Locked Screw Plating of Bicondylar Tibial Plateau Fractures. *Clin Orthop Relat Res*. 2005;439(&NA;):207-214.

113. Kataria H, Sharma N, Kanojia RK. Small wire external fixation for high-energy tibial plateau fractures. *J Orthop Surg* . 2007;15(2):137-143.

114. Bagherifard A, Jabalameli M, Hadi H, et al. Surgical Management of Tibial Plateau Fractures With 3.5 mm Simple Plates. *Trauma Mon*. 2016;21(2):e26733.

115. Lin S, Mauffrey C, Hammerberg EM, Stahel PF, Hak DJ. Surgical site infection after open reduction and internal fixation of tibial plateau fractures. *Eur J Orthop Surg Traumatol*. 2014;24(5):797-803.

116. Dubina AG, Paryavi E, Manson TT, Allmon C, O'Toole RV. Surgical site infection in tibial plateau fractures with ipsilateral compartment syndrome. *Injury*. 2017;48(2):495-500.

117. Faldini C, Manca M, Pagkrati S, et al. Surgical treatment of complex tibial plateau fractures by closed reduction and external fixation. A review of 32 consecutive cases operated. *J Orthop Traumatol*. 2005;6(4):188-193.

© 2019 Norris GR et al. *JAMA Network Open.*

118. Stannard JP, Wilson TC, Volgas DA, Alonso JE. The less invasive stabilization system in the treatment of complex fractures of the tibial plateau: short-term results. *J Orthop Trauma*. 2004;18(8):552-558.

119. Ahearn N, Oppy A, Halliday R, et al. The outcome following fixation of bicondylar tibial plateau fractures. *Bone Joint J*. 2014;96-B(7):956-962.

120. Bobic V, O'Dwyer KJ. Tibial plateau fractures: the arthroscopic option. *Knee Surg Sports Traumatol Arthrosc*. 1993;1(3):239-242.

121. Russell N, Tamblyn P, Jaarsma R. Tibial plateau fractures treated with plate fixation: to lock or not to lock. *Eur J Orthop Surg Traumatol*. 2008;19(2):75.

122. Lee JA, Papadakis SA, Moon C, Zalavras CG. Tibial plateau fractures treated with the less invasive stabilisation system. *Int Orthop*. 2007;31(3):415-418.

123. Manidakis N, Dosani A, Dimitriou R, Stengel D, Matthews S, Giannoudis P. Tibial plateau fractures: functional outcome and incidence of osteoarthritis in 125 cases. *Int Orthop*. 2010;34(4):565-570.

124. Biggi F, Di Fabio S, D'Antimo C, Trevisani S. Tibial plateau fractures: internal fixation with locking plates and the MIPO technique. *Injury*. 2010;41(11):1178-1182.

125. Zura RD, Adams SB Jr, Jeray KJ, et al. Timing of definitive fixation of severe tibial plateau fractures with compartment syndrome does not have an effect on the rate of infection. *J Trauma*. 2010;69(6):1523-1526.

126. Tang X, Liu L, Tu C-Q, et al. Timing of internal fixation and effect on Schatzker IV-VI tibial plateau fractures. *Chin J Traumatol*. 2012;15(2):81-85.

127. Kumar A, Whittle AP. Treatment of complex (Schatzker Type VI) fractures of the tibial plateau with circular wire external fixation: retrospective case review. *J Orthop Trauma*. 2000;14(5):339-344.

128. Egol KA, Su E, Tejwani NC, Sims SH, Kummer FJ, Koval KJ. Treatment of Complex Tibial Plateau Fractures Using the Less Invasive Stabilization System Plate: Clinical Experience and a Laboratory Comparison with Double Plating. *The Journal of Trauma: Injury, Infection, and Critical Care*. 2004;57(2):340-346.

129. Dendrinos GK, Kontos S, Katsenis D, Dalas A. TREATMENT OF HIGH-ENERGY TIBIAL PLATEAU FRACTURES BY THE ILIZAROV CIRCULAR FIXATOR. *J Bone Joint Surg Br*. September 1996. doi:10.1302/0301-620X.78B5.0780710

130. Gaunder CL, Zhao Z, Henderson C, McKinney BR, Stahel PF, Zelle BA. Wound complications after open reduction and internal fixation of tibial plateau fractures in the elderly: a multicentre study. *Int Orthop*. May 2018. doi:10.1007/s00264-018-3940-9

131. Yoon RS, Bible J, Marcus MS, et al. Outcomes following combined intramedullary nail and plate fixation for complex tibia fractures: A multi-centre study. *Injury*. 2015;46(6):1097-1101.

132. Iliopoulos E, Morrissey N, Cho S, Khaleel A. Outcomes of the Ilizarov frame use in elderly patients. *J Orthop Sci*. 2017;22(4):783-786.

© 2019 Norris GR et al. *JAMA Network Open.*

133. Singh K, Bauer JM, LaChaud GY, Bible JE, Mir HR. Surgical site infection in high-energy peri-articular tibia fractures with intra-wound vancomycin powder: a retrospective pilot study. *J Orthop Traumatol*. 2015;16(4):287-291.

134. Lovisetti G, Vulcano E, Bettella L, Tasarib R, Tondolo T, Sala F. Treatment with Circular External Fixation of Bicondylar Tibial Fractures: Potential in Accurate Reduction and Efficacy on Functional Results. *J Knee Surg*. 2018;31(5):459-466.

© 2019 Norris GR et al. *JAMA Network Open.*
